# The Activity of Calcium Glycerophosphate and Fluoride against Cariogenic Biofilms of *Streptococcus mutans* and *Candida albicans* Formed In Vitro

**DOI:** 10.3390/antibiotics12020422

**Published:** 2023-02-20

**Authors:** Thamires Priscila Cavazana, Thayse Yumi Hosida, Caio Sampaio, Leonardo Antônio de Morais, Douglas Roberto Monteiro, Juliano Pelim Pessan, Alberto Carlos Botazzo Delbem

**Affiliations:** 1 Department of Preventive and Restorative Dentistry, School of Dentistry, São Paulo State University (UNESP), Rua José Bonifácio, 1193, Araçatuba 16015-050, SP, Brazil; 2 Postgraduate Program in Health Sciences, University of Western São Paulo (UNOESTE), Rua José Bongiovani, 700—Cidade Universitária, Presidente Prudente 19050-920, SP, Brazil

**Keywords:** biofilms, dental caries, phosphates, fluorides, calcium

## Abstract

This study evaluated the effects of calcium glycerophosphate (CaGP), with or without fluoride (F), on dual-species biofilms of *Streptococcus mutans* and *Candida albicans*. The biofilms were treated three times with 0.125, 0.25, and 0.5% CaGP solutions, with or without 500 ppm F (NaF). Additionally, 500 and 1100 ppm F-solutions and artificial saliva served as controls. After the final treatment, the microbial viability and biofilm structure, metabolic activity, total biomass production, and the composition of the extracellular matrix composition were analyzed. Regardless of the presence of F, 0.25 and 0.5% CaGP promoted a higher biomass production and metabolic activity increase than the controls (*p* < 0.05). F-free CaGP solutions reduced bacterial cell population significantly more than the 500 ppm F group or the negative control (*p* < 0.05). All the groups reduced the proteins, and 0.5% CaGP combined with F led to the highest reduction in the carbohydrate and nucleic acids content of the extracellular matrix (*p* < 0.05). It can be concluded that CaGP alone affected the number of bacterial cells and, when combined with F, reduced its production of biomass, metabolic activity, and the expression of the extracellular matrix components.

## 1. Introduction

While the prevalence of dental caries has reduced worldwide, this disease remains an important public health concern, given its impact on the oral health of the overall population [1]. Sugar-biofilm interactions are regarded as the main etiologic factor underlying dental caries [2,3], and the bacterium *Streptococcus mutans* is the most important species involved in the development of this disease [4]. In addition to *S. mutans*, several other species, such as *Candida albicans,* participate in the development of dental caries. This microorganism is found in the oral environment, and its contribution to the onset and progression of caries has been thoroughly emphasized in recent years [5,6].

The use of fluoridated products is considered one of the main reasons for the reduced prevalence and incidence of caries over the years [7]. However, given that approximately 80% of the total daily fluoride (F) intake by toddlers is through the ingestion of F-dentifrice, the use of such products has been associated with the development of dental fluorosis [8,9]. Studies have examined F-product supplementation with polyphosphate salts to help reduce F intake without compromising the protective effect of the ion. Calcium glycerophosphate (CaGP), an organic polyphosphate, markedly enhances enamel de- and remineralization favoring the prevention of dental mineral loss, which is essential for caries control [10,11]. However, the evidence regarding the effects of CaGP in dental biofilms remains scarce, primarily focusing on the changes in the ionic composition caused by this polyphosphate, related to the saturation of the biofilm with the minerals of the dental structure that can prevent the breakage of hydroxyapatite crystals in the tooth [12,13].

Recent data have shown that biofilms of *S. mutans* and *C. albicans* are affected by inorganic polyphosphates [14,15], whereas the effects of CaGP on these biofilms related to caries disease are unknown. Thus, this study aims to evaluate the effects of CaGP, with or without F, on the viability, metabolism, biomass production, and structure and composition of the extracellular matrix of dual biofilms formed by *S. mutans* and *C. albicans*. The null hypothesis is that CaGP, whether it is combined with F or not, does not affect the physiological and structural parameters of the biofilms.

## 2. Results

### 2.1. Evaluation of Extracellular Matrix Composition

**Figure 1:**
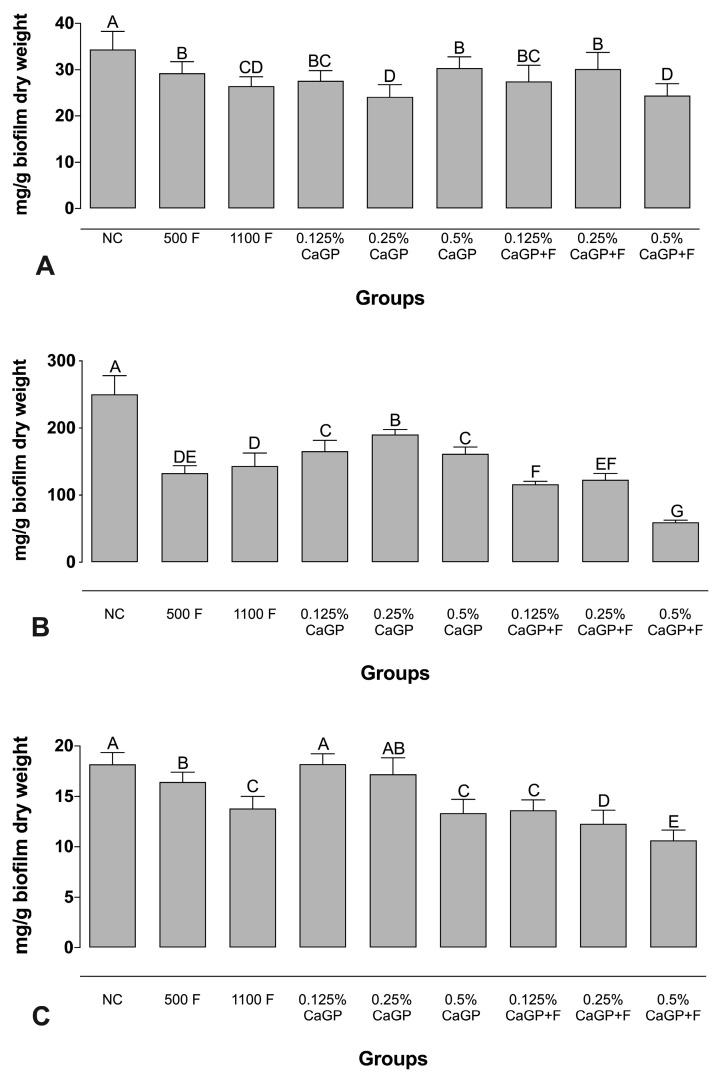
Mean values of (**A**) Protein, (**B**) Carbohydrate, and (**C**) Nucleic acids from the extracellular matrix of dual-species biofilms obtained after treatment with different concentrations of CaGP, with or without an association with F. Error bars denote the standard deviations of the means. Different upper-case letters symbolize statistical differences among the groups (1-way ANOVA, Fisher’s LSD test, *p <* 0.05; n = 9). NC: negative control (untreated biofilms); CaGP: calcium glycerophosphate; F: fluoride.

Regarding the amount of protein, all treatments interfered with these components of the biofilm, and lower values were observed for groups treated with 0.25% and 0.5% CaGP + F, with no significant difference compared to the 1100 ppm F group (*p* > 0.05; Figure 1). No significant differences were observed for the biofilms treated with CaGP at 0.125% and 0.25% with F, compared to 500 ppm F (*p* > 0.05; Figure 1). As for carbohydrates, all the treatments reduced the amount found in the biofilms and we observed that CaGP at 0.5% + F promoted the highest reduction (~23% lower than the NC and ~42% lower than the 1100 ppm F group). In terms of nucleic acid content, biofilms exposed to 1100 ppm F showed lower concentrations than those exposed to 500 ppm F. CaGP at higher concentrations led to greater reductions than the NC, while lower values of nucleic acids were found in groups treated with CaGP associated with F (Figure 1).

In the analyzed biofilm, treatments with CaGP significantly reduced the carbohydrates in comparison to the NC group (Figure 1B). The CaGP concentrations in the treatment with the highest concentrations promote values of the components (proteins, carbohydrates and nucleic acids), statistically similar or with a greater reduction when observed with lower concentrations (Figure 1).

### 2.2. Quantification Assays

**Figure 2:**
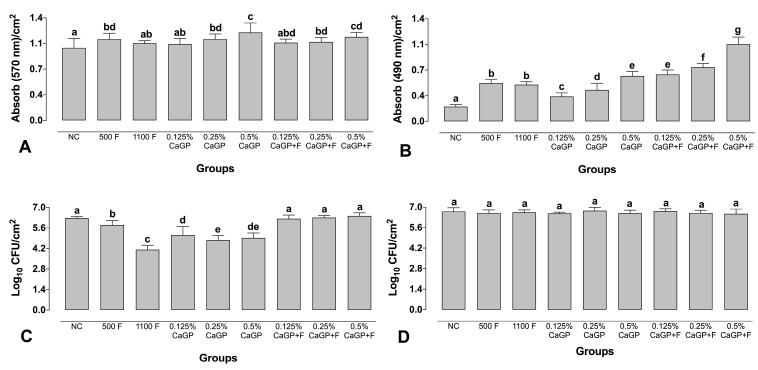
Absorbance values per cm^2^ obtained for quantification of Total biomass (**A**), Metabolic activity (**B**), Logarithm of colony-forming units per cm^2^ for *Streptococcus mutans* (**C**) *Candida albicans* (**D**) in dual-species biofilms. NC: negative control (untreated biofilms); CaGP: calcium glycerophosphate; F: fluoride. Error bars denote the standard deviations of the means. Different letters symbolize statistical differences among the groups (1-way ANOVA, Fisher’s LSD test, *p <* 0.05; n = 9).

Treatment with 500 ppm F alone and CaGP at 0.25 and 0.5% (with or without F) led to an increase in the biofilm biomass compared to the NC (*p* < 0.001; Figure 2A). The highest biomass values were observed for biofilms treated with 0.5% (1.20 abs/cm^2^) and 0.5% associated with F (1.14 abs/cm^2^). Biofilms treated with 1100 ppm F and those exposed to 0.125% CaGP (with or without F) did not differ from the NC group (*p* > 0.05; Figure 2A).

All treatments promoted a significant increase in the metabolic activity of the biofilms compared to the NC, and the solutions containing only F (500 ppm F and 1100 ppm F) did not differ from each other (*p* > 0.05; Figure 2B). A dose-response effect was observed in the groups treated using CaGP-containing solutions; the higher the concentration of CaGP, the higher the metabolic activity of the biofilms. Groups treated with CaGP associated with F showed higher metabolism values than their counterparts without F (Figure 2B).

All the CaGP-containing solutions associated with F did not lead to significant reductions in the CFUs of *S. mutans* compared to the NC (*p* > 0.05; Figure 2C), while treatments with CaGP alone significantly decreased the CFUs compared to the NC (*p* < 0.001; Figure 2C). All the CaGP solutions without F led to greater reductions in the CFUs of *S. mutans* compared to the groups exposed to 500 ppm F, while the highest reduction was observed for the 1100 ppm F group. For *C. albicans*, exposure to any of the treatment solutions did not significantly affect the number of CFUs (Figure 2D).

### 2.3. Structural Analysis of Biofilms

**Figure 3:**
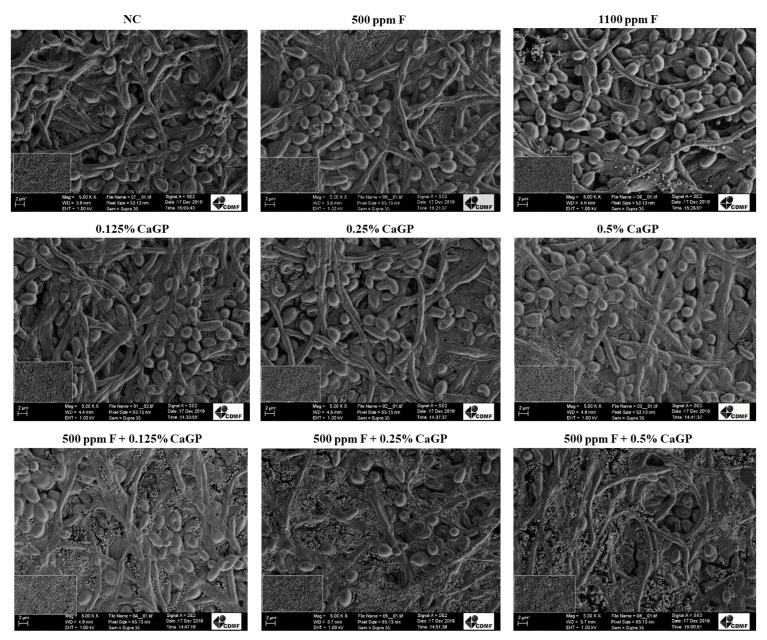
Scanning electron microscopy images of dual-species biofilms of *Candida albicans* and *Streptococcus mutans* after treatments with the experimental solutions. Magnification: 5000×. Bars: 2 mm. NC: negative control (untreated biofilms); CaGP: calcium glycerophosphate; F: fluoride.

In general, no structural differences were noted among biofilms treated with the different solutions, and all biofilms exhibited cocci attached to yeasts and hyphae, forming dense networks (Figure 3). The biofilms treated with 1100 ppm F presented fewer bacterial cells than the other groups (Figure 3).

## 3. Discussion

F is considered an important agent for controlling dental caries worldwide [16]; however, excessive F ingestion can lead to dental fluorosis. Therefore, strategies to reduce systemic F exposure without compromising its protective effect have been examined. In this sense, one of the possibilities is lowering F concentrations in F-containing products and supplementing them with CaGP, which is known to promote a similar protective effect to a conventional dentifrice (1100 ppm F) on enamel de- and remineralization [10,11]. In the present study, CaGP, with or without F, influenced parameters related to the extracellular composition, biofilm metabolism, and biomass production, thus partially rejecting the study’s null hypothesis.

According to Lynch [12], exposure to CaGP leads to higher levels of calcium (Ca) and phosphate (P) in biofilms, which is the most likely explanation for the anti-caries potential of this polyphosphate. The ability of microorganisms within biofilms to detect changes in the components of the medium is an important task that allows pathogens to efficiently adjust to the environment, triggering the expression of genes responsible for their resistance [17]. Furthermore, tolerance to Ca can play a crucial role in cariogenic bacteria [18] since this ion alters the cellular metabolism of microorganisms and their ability to detect and respond to environmental concentrations, which is an important aspect of microbial pathogenesis [19].

Bacterial influx systems for cations contribute to pathogenesis, and efflux systems have primarily been characterized in contaminated environmental sites. High calcium concentrations in the medium are toxic to gram-positive bacteria, and their survival depends on Ca^2+^ exporters [19]. Due to the influx of Ca channels, the levels of this cation can increase in the cell cytosol [20]. To ensure this efflux, the pumps require ATP to maintain the appropriate intracellular concentrations [21]. The consequences of high Ca^2+^ growth include the activation of autolysis and the induction of competence [22]. The increased Ca concentration in the medium triggers a change in bacterial nucleic acid and cell lysis. This modification in the genetic material is related to its ability to inhibit Ca uptake. Bacteria that can capture exogenous genetic material with competence can overcome lysis and survive in environments with high concentrations of Ca [22]. These changes related to Ca and bacteria were observed in the results. Biofilms treated only with CaGP showed a decrease in the number of *S. mutans* and higher nucleic acid values in the medium compared to the same CaGP concentrations associated with 500 ppm F. Thus, the treatments with high Ca concentrations, as demonstrated previously [13], may have induced cellular changes, resulting in cell lysis and release of genetic material that may be competent for survival in a high-Ca environment.

The response of *C. albicans* to the increase in Ca concentrations may be reflected in the increased metabolic activity of the biofilm that occurred when the treatment contained only CaGP, which showed a decrease in *S. mutans* cells. In this microorganism, Ca^2+^ channels open and allow Ca^2+^ to passively enter the cytosol owing to the concentration gradient from the extracellular space or intracellular stores. The Ca^2+^/H^+^ antiporters are the driving force of protons, and Ca^2+^ pumps use ATP to move Ca^2+^ against a concentration gradient outside the cytosol to maintain ideal internal levels. *C. albicans* also has Ca-ATPase in the vacuolar membrane to overcome the toxic effect of cations, storing its excess in vacuoles [23], which is essential for its growth in media with a high Ca^2+^ content [24]. Excess external Ca^2+^ affects the formation of hyphae, resulting in the formation of pseudo-hyphae and a decrease in the virulence of this fungus [25].

Furthermore, F alone also led to increased metabolic activity in biofilms, as reported previously [26]. The groups that received CaGP combined with F presented the highest metabolic activity, which can be explained by the cumulative effect of Ca (from CaGP) and F on such microorganisms. CaGP associated with F did not interfere with the number of *S. mutans* cells compared to the NC. This may be related to CaF_2_ formation (resulting from Ca and F availability), thus reducing the availability of Ca in the medium. Additionally, this interaction also interfered with the amount of F in the environment, which led to the effect observed in the 500 ppm F group without polyphosphate, supporting this hypothesis. Regarding *C. albicans*, no difference in the CFUs number was observed, possibly because this fungus was not affected by the number of ions provided by the CaGP and CaGP + F treatments.

Treatment with a high Ca content increases the calcium bound to the surface of dental biofilm bacteria [27]. Calcium interacts with surface proteins and forms ionic bridges between negatively charged macromolecules, thereby improving cell aggregation and strengthening biofilm matrices. The proximity of neighboring cells reportedly depends on the calcium bridge [28], and the presence of Ca leads to a connection between molecules [29]. This change in biofilm composition may justify the increase in biomass production found for biofilms treated with CaGP by 0.25 and 0.5% CaGP, regardless of the presence of F, compared with NC. It is suggested that the binding of Ca and microorganisms can retain new compounds, thus increasing biofilm biomass. This hypothesis is in line with the quantitative results of the present study, considering that the increase in biomass was not accompanied by an increase in the number of cells or components of the extracellular matrix. Additionally, a structure with fewer spaces between cells, observed in the SEM analysis for these groups, possibly resulted from calcium bridges within the biofilm.

Structural rigidity for protection from the external environment and controlling gene regulation and nutrient adsorption are essential functions of the extracellular matrix [30]. Sucrose is essential for extracellular matrix production, thus affecting biofilm growth and adhesion [31]. For this reason, it was added to saliva in the present study. The treatment time of 1 minute with the solutions is due to the simulation of the time it takes a person to brush their teeth [14,15]. Treatment with CaGP and F effectively reduced carbohydrates, proteins, and nucleic acids in the extracellular matrix. Similar results were reported in a previous study that tested the effect of inorganic polyphosphate on dual-species biofilms of *S. mutans* and *C. albicans* [14]. An in situ study also showed a significant reduction in the concentration of extracellular polymeric substances (EPS) in biofilms exposed to CaGP and F [10], thus reinforcing the present findings.

The presence of water-insoluble glucans in the biofilm matrix is a relevant factor for the pathogenicity of biofilms related to dental caries [32]. These polysaccharides hinder the penetration of drugs and create acidic areas, favoring the demineralization of dental surfaces [33]. In this sense, carbohydrate reduction is a favorable aspect of controlling caries. Lower protein and nucleic acid values also play an important role in the formation of dental caries, considering their involvement in the adhesion of the biofilm to surfaces, allowing cell communication, horizontal transference of genes between cells, and other functions of the extracellular matrix [34].

The data evaluated in the present study have already been confirmed in the literature using other types of polyphosphates with or without an association with F [14,15]. It is known that the biofilm components, regardless of the phosphate used, increased the reduction with the treatments. Comparing the results, only CaGP increased the metabolic activity and biofilm biomass, possibly because it is a source of Ca, as discussed in the previous paragraphs. None of the phosphates studied could interfere with the number of *C. albicans* [14,15].

Despite these promising trends, it is critical to highlight that the dual-species biofilms addressed in this study do not fully represent polymicrobial consortia in the oral environment. Further investigations addressing multispecies biofilms under dynamic conditions are recommended to confirm the results obtained in the present study.

## 4. Materials and Methods

### 4.1. Biofilm Formation

Strains from the American Type Culture Collection (ATCC) of *C. albicans* (ATCC 10231) and *S. mutans* (ATCC 25175) were used in this study. *S. mutans* colonies grown on brain heart infusion agar (BHI Agar; Difco, Le Pont de Claix, France) were added to 10 mL of BHI broth (Difco) and incubated (statically) overnight in 5% CO_2_ at 37 °C. For *C. albicans*, colonies previously cultured on Sabouraud dextrose agar (SDA; Difco) were added to 10 mL of Sabouraud dextrose broth (Difco) and incubated (aerobically) overnight at 120 rpm and 37 °C. The cells were centrifuged (6876× *g*, 5 min), and the pellets were washed twice with 10 mL of NaCl (0.85%). The number of cells was adjusted in artificial saliva (AS) to 1 × 10^7^ cells/mL of *C. albicans* using a Neubauer counting chamber, while the number of *S. mutans* was spectrophotometrically (640 nm) adjusted to 1 × 10^8^ cells/mL. Dual-species biofilms were formed in flat-bottomed microtiter plates by adding the microbial suspensions (1 × 10^7^ cells mL^−1^ for *C. albicans* + 1 × 10^8^ cells mL^−1^ for *S. mutans*) in AS [14]. The plates were incubated in 5% CO_2_ at 37 °C for 96 h. Every 24 h, half of the well contents were replenished with AS by gently aspiring half of the contents with a pipette and renewing it with the same amount of fresh AS. The AS was prepared according Cavazana et al. [35] as follows: for 1 l demi-water: sucrose (4 g), yeast extract (2 g), bacteriological peptone (5 g), mucin type III (1 g), NaCl (0.35 g), CaCl_2_ (0.2 g), and KCl (0.2 g), at pH 6.8. All components for the AS preparation were purchased by Sigma-Aldrich (St Louis, MO, USA).

### 4.2. Treatment of the Biofilms

The concentrations of CaGP (Sigma-Aldrich, St Louis, MO, USA) adopted in this study were determined according to previous data [11], resulting in the following experimental groups: CaGP at 0.125, 0.25, and 0.5%, with or without 500 ppm F. In addition, solutions containing 500 and 1100 ppm F were evaluated, while pure AS was tested as a negative control (NC).

The biofilms were treated after 72, 78, and 96 h from their formation, resulting in three treatments [14,36]. All the AS was gently removed by aspiration, and the biofilms were exposed to the experimental solutions for 1 min by gently pipetting the solutions into the wells [14]. Next, the solutions were removed from the wells, and the biofilms received fresh AS [14]. After the last treatment (96 h after the beginning of formation), the biofilms were washed with 0.85% NaCl, and the quantitative and qualitative experiments were performed [14].

### 4.3. Evaluation of Extracellular Matrix Composition

For this assay, dual-species biofilms were grown in six-well plates (Costar^®^ #3516, Corning Inc., Corning, NY, USA) containing 4 mL of the microbial suspension, as previously detailed. After the last treatment, the biofilms were resuspended in 0.85% NaCl, scraped from the wells, and the liquid phase of the extracellular matrix was extracted by sonication (for 30 s at 30 W) [37]. Protein determination of the extracellular matrix was performed by the bicinchoninic acid method (Kit BCA; Sigma-Aldrich), using bovine serum albumin as the standard [37]. The carbohydrate content was quantified, as detailed by Dubois et al. [38], with glucose as the standard. For the evaluation of the nucleic acid content, 1.5 mL of the liquid phase of the extracellular matrix was spectrophotometrically analyzed (at 260 and 280 nm) in a Nanodrop Spectrophotometer (EON Spectrophotometer of EON, Biotek, Winooski, VT, USA) [39]. Protein, carbohydrate, and nucleic acid values were expressed as mg/g dry weight of biofilm.

### 4.4. Quantification Assay

For the quantification assays, the dual-species biofilms of *S. mutans* and *C. albicans* were grown in 96-well plates (Costar^®^ #3595, Corning Inc., Corning, NY, USA). The biofilm biomass was evaluated by the crystal violet (CV) staining assay, as detailed by Monteiro et al. [40]. For this, the biofilms were fixed with 99% methanol (Sigma-Aldrich) for 15 min, stained for 5 min with 1% CV (Sigma-Aldrich), and de-stained through exposure to 33% acetic acid (Sigma-Aldrich). Absorbance values were read (570 nm) and represented as a function of the area of the wells (absorbance/cm^2^). AS without microbial cells was used as blank.

Investigating the effect of the treatment solutions on the metabolic activity of the biofilms was performed by the 2,3-bis(2-methoxy-4-nitro-5-sulfophenyl)-5-[(phenylamino) carbonyl]-2H-tetrazolium hydroxide (XTT; Sigma-Aldrich) reduction method [40]. Briefly, XTT and phenazine methosulphate (Sigma-Aldrich) solutions were combined and pipetted into the wells, and the plates were then incubated at 37 °C (for 3 h at 120 rpm), protected from light. The absorbance values were measured at 490 nm (absorbance/cm^2^). AS without microbial cells was used as blank.

Counting of colony-forming units (CFUs) was performed to evaluate the number of cultivatable cells. After the last treatment, the biofilms were resuspended in NaCl (0.85%) and scraped from the wells. Biofilm suspensions were then serially diluted in 0.85% NaCl and plated on CHROMagar Candida (Difco) for counting *C. albicans* and on BHI agar supplemented with amphotericin B (7 µg/mL; Sigma-Aldrich), for counting *S. mutans*. Agar plates were incubated for 24–48 h at 37 °C, and the number of CFUs was expressed as log^10^ CFU/cm^2^ [14].

### 4.5. Structural Analysis of the Biofilm

To evaluate the biofilm structure, the dual-species biofilms were formed in 24-well plates (Costar^®^ #3524, Corning Inc., Corning, NY, USA) and evaluated by scanning electron microscopy (SEM). In brief, dual-species biofilms of *C. albicans* and *S. mutans* were formed in 24-well plates and exposed to the experimental solutions, as previously described. After the last treatment, the wells were gently washed with NaCl (0.85%), and the biofilms were dehydrated using the following series of ethanol concentrations: 70% for 10 min, 95% for 10 min, and 100% for 20 min, followed by air drying for 20 min [41]. Next, the bottom of each well was cut with a flame-sterilized scalpel blade (number 11, Solidor, Lamedid Commercial and Services Ltd., Barueri, Brazil), and the biofilms were coated with gold and evaluated using an electron microscope (S-360 microscope, Leo, Cambridge, MA, USA).

### 4.6. Statistical Analysis

All assays were performed in triplicate on three different occasions (n = 9). Data passed normality and homogeneity tests (Shapiro-Wilk) and were submitted to ANOVA, followed by Fisher’s LSD post hoc test. Statistical analysis was performed using SigmaPlot 12.0 software (Systat Software Inc., San Jose, CA, USA), adopting *p* < 0.05.

## 5. Conclusions

Based on the above results, it can be concluded that CaGP alone affected the number of *S. mutans* cells. Also, CaGP, in combination with F, led to reductions in the biofilms’ production of biomass and metabolic activity, as well as substantially reducing the components of the extracellular matrix. The data presented in this study elucidate the way that CaGP, with or without F, acts on cariogenic-related dual-species biofilms of *S. mutans* and *C. albicans* formed in vitro.

## Data Availability

The data presented in this study are available on request from the corresponding author.
